# Solubility of Naproxen in Polyethylene Glycol 200 + Water Mixtures at Various Temperatures

**Published:** 2015

**Authors:** Vahid Panahi-Azar, Shahla Soltanpour, Fleming Martinez, Abolghasem Jouyban

**Affiliations:** a *Liver and Gastrointestinal Diseases Research Center, Tabriz University of Medical Sciences, Tabriz, Iran.*; b *Faculty of Pharmacy, Zanjan University of Medical Sciences, Zanjan, Iran.*; c *Grupo de Investigaciones Farmacéutico-Fisicoquímicas, Departamento de Farmacia, Universidad Nacional de Colombia, Bogotá D.C., Colombia. *; d *Drug Applied Research Center and Faculty of Pharmacy, Tabriz University of Medical Sciences, Tabriz, Iran.*; e *Pharmaceutical Engineering Laboratory, School of Chemical Engineering, College of Engineering, University of Tehran, Tehran, Iran. *

**Keywords:** Thermodynamic analysis, Solubility, Naproxen, Jouyban–acree model

## Abstract

The solubility of naproxen in binary mixtures of polyethylene glycol 200 (PEG 200) + water at the temperature range from 298.0 K to 318.0 K were reported. The combinations of Jouyban-Acree model + van’t Hoff and Jouyban-Acree model + partial solubility parameters were used to predict the solubility of naproxen in PEG 200 + water mixtures at different temperatures. Combination of Jouyban-Acree model with van’t Hoff equation can be used to predict solubility in PEG 200 + water with only four solubility data in mono-solvents. The obtained solubility calculation errors vary from ~ 17 % up to 35 % depend on the number of required input data. Non-linear enthalpy-entropy compensation was found for naproxen in the investigated solvent system and the Jouyban−Acree model provides reasonably accurate mathematical descriptions of the thermodynamic data of naproxen in the investigated binary solvent systems.

## Introduction

Solubility of drugs is an important issue in the pharmaceutical area, because it permits the scientist to choose the best solvent or solvent mixture for dissolving a drug or combination of drugs. Solutions of the drugs could be used to measure the purity of drug bulks, prepare a liquid formulation and/or extract an ingredient from a synthetic mixture or a natural source. So, it is important to determine the solubility of pharmaceutical compounds systematically. Also, using solubility data at various temperatures thermodynamic analysis and better understanding of molecular mechanisms could be investigated.

Polyethylene glycols (PEGs) are the polymers that prepared from ethylene oxide and water with the general formula of H (OCH_2_CH_2_) _n_OH. In this formula n is the average number of repeated oxyethylene groups which is from 4 to 180. The numeric suffix beside the abbreviation, PEG, indicates the average molecular weight. Solubility of PEGs in water is an important property, which makes them suitable for using in different applications. Liquid PEGs up to 800 are freely miscible with water. The liquid PEGs have a slightly bitter taste, but it could be easily adjusted by suitable additives (sweeteners) and solid PEG grades show a neutral taste (1). The compounds with lower molecular weight (up to 800) are colorless, odorless viscous liquids with a freezing point from -10ºC (diethylene glycol), while polymerized compounds with higher molecular weight (more than 1,000) are wax like solids with melting point up to 67ºC for n = 180 (2). 

Naproxen (NAP) is a nonsteroidal anti-inflammatory drug, which is used for its pain relieving and anti-inflammatory effects. It possesses low aqueous solubility and salt form of NAP is used to improve its solubility. Once NAP or its salt form is dissolved in biological fluids, both forms have the same biological properties. NAP solubility has been studied in ethanol + water cosolvent mixtures as molecular (non-dissociate) form and sodium salt (3, 4) and in propylene glycol + water mixtures as molecular form (5). In this work solubility of NAP in PEG 200 + water mixtures at various temperatures was measured and the data is used to derive some classical dissolution thermodynamic properties, including enthalpy-entropy compensation analysis.

## Experimental

*Chemicals*

NAP (98.5 % w/w) was purchased from Mahban Chemi (Tehran, Iran). The purity of NAP was checked by measuring the melting point range (427K to 431K) and by comparing the measured solubilities in mono-solvents with the corresponding data from the literatures (3). PEG 200 (99.0 % w/w) was purchased from Merck (Germany) and ethanol (93.5 % w/w) from Jahan Alcohol Teb (Arak, Iran). Double distilled water was used for preparation of the solutions.

*Apparatus and procedures*

All binary mixtures were prepared with appropriate mass fractions. Various methods were reported for determination of drug’s solubility (6). The solubility of NAP was determined using shake flask method of Higuchi and Connors (7) by equilibrating an excess amount of the solid in the prepared binary solvent mixtures using a shaker (Behdad, Tehran, Iran) placed in an incubator equipped with a temperature-controlling system at different temperatures with the uncertainty of 0.2K (Nabziran, Tabriz, Iran) for 3 days to reach the equilibrium at 298K. After solubility determination and density measurement at 298K, the remaining solutions containing excess solid were placed at 303K for 2 day and the measurements were carried out and the same procedure was repeated for the rest of temperatures. The solutions were filtered using hydrophilic Durapore filters (0.45μm, Millipore, Ireland) and after diluting with ethanol, the absorbance of these solutions were recorded at 271 nm using a UV-vis spectrophotometer (Beckman DU-650, Fullerton, USA). Calibration graph is the molar absorptions of NAP ranging from 199778.1* ε*/ (L.mol^-1^ cm^-1^) to 1667709.2 *ε*/ (L.mol^-1^ cm^-1^) and mean relative standard deviations (RSDs) of three repetitive experiments is 2.6 %. Concentrations of the dilute solutions that were determined by UV absorbance of each experimental data are an average of at least three experimental measurements. Densities of the saturated solutions were determined using a 5 mL pycnometer.

## Results and Discussions

Table 1 shows the mass fraction composition of the binary solvent mixtures, densities of the saturated solutions, and solubility of NAP at investigated temperatures. The minimum solubility of NAP (among investigated solvent systems) is observed in aqueous solution at 298.2K and addition of PEG 200 to the aqueous solutions and also increasing the temperature resulted in solubility enhancement. NAP mole fraction solubility values in neat water presented in Table 1 are in good agreement with the ones previously reported at temperatures from 25 to 40°C, i.e. 5.13 10^-6^ (at 25°C), 5.91 10^-6^ (at 30°C), 6.60 10^-6^ (at 35°C), and 7.68 10^-6^ (at 40°C) (3).

**Table 1:** Mole fraction solubility of naproxen in various mass fractions (*m*_1_) of PEG 200 (1) + water (2) mixtures at several temperatures (ºC).

***m***_1_	**25.0**	**30.0**	**35.0**	**40.0**	**45.0**
0.000	5.12 x 10^-6^	6.13 x 10^-6^	6.94 x 10^-6^	8.02 x 10^-6^	8.56 x 10^-6^
0.100	1.36 x 10^-5^	2.35 x 10^-5^	3.39 x 10^-5^	6.16 x 10^-5^	8.46 x 10^-5^
0.200	3.85 x 10^-5^	5.11 x 10^-5^	8.25 x 10^-5^	1.46 x 10^-4^	2.28 x 10^-4^
0.300	1.93 x 10^-4^	3.18 x 10^-4^	4.23 x 10^-4^	5.24 x 10^-4^	6.82 x 10^-4^
0.400	3.85 x 10^-4^	5.08 x 10^-4^	6.77 x 10^-4^	1.06 x 10^-3^	1.40 x 10^-3^
0.500	8.43 x 10^-4^	1.30 x 10^-3^	1.58 x 10^-3^	1.95 x 10^-3^	2.32 x 10^-3^
0.600	2.31 x 10^-3^	3.08 x 10^-3^	3.68 x 10^-3^	4.44 x 10^-3^	5.03 x 10^-3^
0.700	3.81 x 10^-3^	5.63 x 10^-3^	7.78 x 10^-3^	1.01 x 10^-2^	1.21 x 10^-2^
0.800	7.77 x 10^-3^	1.10 x 10^-2^	1.45 x 10_-2_	2.00 x 10^-2^	2.50 x 10^-2^
0.900	1.23 x 10^-2^	1.79 x 10^-2^	2.45 x 10^-2^	3.22 x 10^-2^	4.06 x 10^-2^
1.000	1.95 x 10^-2^	2.74 x 10^-2^	4.43 x 10^-2^	6.06 x 10^-2^	8.50 x 10^-2^

The general form of the model for representing the solubility of a solute in binary solvent mixture at various temperatures is (8):

 Equation (1) Log Xm,TSat=m1.logX1,TSat+m2.logX2,TSat+m1.m2T. ∑i=02Ji.(m1-m2)i .

in which $X_{m,T}^{\mathrm{Sat}}$ is the solute mole fraction solubility in the solvent mixtures at temperature *T *(K), $m_{1}$ and $m_{2}$ are the mass fractions of the solvents 1 and 2, respectively, $X_{1,T}^{\mathrm{Sat}}$and $X_{2,T}^{\mathrm{Sat}}$ are the mole fraction solubility of the solute in the neat solvents 1 and 2, respectively, and $J_{i}$ are the constants of the model computed by regression analysis. When all generated solubility data points were fitted to Equation 1, the obtained model is:

Log Xm,TSat=m1.logX1,TSat+m2.logX2,TSat+635.634m1m2T-225.363m1m2(m1-m2)T

 Equation (2)

which is correlated the solubility data with the mean relative deviation (MRD) of 18.0 (± 21.2) % (N=55). The MRD is calculated using:

$\mathrm{MRD}=\frac{100}{N}\left(\frac{X_{m}^{\mathrm{Calculated}}-X_{m}^{\mathrm{Observed}}}{X_{m}^{\mathrm{Observed}}}\right)$

 Equation (3)

in which N is the number of data points in each set. Considering wide solubility range of NAP in the investigated solvent system (from aqueous mole fraction solubility of 5.12 x 10^-6^ at 25ºC to 8.5 x 10^-2^ in neat PEG 200 at 45ºC), the produced MRD could be considered as acceptable error level. In addition, the accepted error level for solubility correlation in the literature is < 30 % (8-10). Equation 2 could be used to evaluate the generated solubility data to detect the possible outliers. In addition, the computed model constants enable us to predict the solubility of NAP in all possible solvent compositions of PEG 200 + water mixtures at temperatures of interest using interpolation technique.

A main limitation of Equation 1 for its practical applications in the pharmaceutical industry is computing the model constants that require a number of experimental solubility data of the solute in the binary solvent mixtures. There are some limitations in measuring large number of solubility data points especially in early stages of drug discovery where only few milligrams of a drug candidate is available and the medicinal chemist should determine some other physico-chemical properties required for further assessment of drug activity. In addition, in drug development and formulation stages, R & D departments prefer to use in-silico prediction models instead of costly experimental trial and error approaches. When accurate in-silico methods are not available, the researchers rely on reducing the number of required experimental data points. As noticed above, Equation 1 should be trained by experimental data points for predictive uses. Various procedures have been proposed to cover this limitation and reduce the number of required experimental data points. It has been shown that the model constants of Equation 1 could be computed using the solubility data at 298.2K and then the solubility data at other temperatures could be predicted using experimental solubility data in the mono-solvents at each temperature of interest (11-13) which reduces the number of experimental data points from 11 points (considering 0.1 solvent fraction intervals) to just two data points at each temperature after training process of the model. To show the applicability of this method to predict solubility data of NAP in PEG 200 + water mixtures, the model is trained using solubility data at 298.2K, and the rest of data points were predicted using the trained model by employing the solubility data in the mono-solvents, i.e. $X_{1,T}^{\mathrm{Sat}}$and$X_{2,T}^{\mathrm{Sat}}$. The MRD for the predicted data points is 18.0 (± 19.9) % (N = 36). There is no significant difference between back-calculated error and that of the predicted data points using this computational method (t-test, p > 0.05). As another attempt, in a previous work, the Jouyban-Acree model was trained using the solubility data of different drugs in PEG 400 + water mixtures (14). In this version, it was assumed that the model constants are independent from the nature of solutes. The trained model for PEG 400 + water mixtures was used to predict the solubility of drugs in PEG 200 + water mixtures with acceptable prediction error (15). This version of the model requires the solubility in PEG 200 and water at various temperatures of interest. The trained version of the Jouyban-Acree model for aqueous binary mixtures of PEG 400 at different temperatures is (14):

Log Xm,TSat=m1.logX1,TSat+m2.logX2,TSat+394.82m1m2T-355.28m1m2m1-m2T+388.89m1m2m1-m2m1-m22T

 Equation (4)

and is used to predict the solubility of NAP in PEG 200 + water mixtures with the MRD of 25.8 (± 20.5) % (N = 45). Using this approach, there is no need to employ any data point in PEG 200 + water mixtures.

Solubility of drugs in mono-solvents at different temperatures can be calculated using van’t Hoff approach (16). The required experimental data are solubilities at the lowest and highest temperatures (${\mathrm{log X}}_{1,T}^{\mathrm{Sat}}$). 

$\log X_{T}^{\mathrm{Sat}}=A+\frac{B}{T}$

Equation (5)

where A and B are the model constants calculated by regression method. Using Equation 5 in the computations may further reduce the number of experiments required in the prediction procedures. A combination of Jouyban-Acree and van’t Hoff model was used for solubility prediction of compounds in mono- and mixed solvents at different temperatures (17):

Log Xm,TSat=m1A1+B1T+m2A2+B2T+m1.m2T. ∑i=02Ji.(m1-m2)i

Equation (6)

Equation 6 is trained using the generated solubility data and the obtained model is:

Log Xm,TSat=m16.940-2557.410T+m23.512-2662310T+593.725m1m2T-209.81m1m2m1-m2T

Equation (7)

which back-calculated the solubility data with the MRD of 17.4 (± 17.3) % (N = 55). This version of the model could be trained using the solubility data at two temperatures, *i.e*. the lowest and the highest temperature of interest, and then could be used to predict the solubility in the mono- and mixed solvents. When Equation 6 is trained using solubility data at 298.2 and 318.2 K, the solubility of NAP in PEG 200 + water at 303.2, 308.2 and 313.2K was predicted with the MRD of 18.2 (± 17.5) % (N = 33). When Equation 6 is trained using a minimum number of 10 data points, *i.e*. the solubility data of *m*_1_=0.0, 0.3, 0.5, 0.7, and 1.0 at 25ºC and the same mixtures at 45ºC, the rest of data points were predicted with the MRD of 22.0 (± 23.0) % (N = 45).

Equation 4 is a specific model to predict the solubility of drugs in PEG 400 (and also PEGs 200 or 600) and water binary mixtures at various temperatures. In some cases, these solvent systems are not able to dissolve a desired amount of drug in a given volume of the solvent system, and employing other cosolvents is required to overcome the solubility problem. For such cases, a generally trained model which is a combination of the Jouyban–Acree model and Hansen partial solubility parameters was proposed for predicting the solubility of various drugs in different binary mixtures of cosolvent + water as (18):

Log Xm,T=m1logX1,T+m2logX2,T+m1m2T0.606δps(δp1-δp2)2+0.013δhs(δh1-δh2)2+m1m2(m1-m2T0.696δds(δd1-δd2)2+0.013δhs(δh1-δh2)2+m1m2(m1-m22T9.277δds(δd1-δd2)2+0.461δhs(δh1-δh2)2+

Equation (8)

Where$\delta_{\mathrm{ds}}$ , $\delta_{\mathrm{ps}}$and $\delta_{\mathrm{hs}}$ are the partial solubility parameters of the solute,$\delta_{d}$, $\delta_{h}$and $\delta_{p}$ are the partial solubility parameters of solvents and subscripts 1 and 2 denote cosolvent and water, respectively. Equation 8 is able to predict the solubility in aqueous mixtures of pharmaceutical cosolvents. The produced MRD for the predicted solubilities using Equation 8 is 33.4 (± 23.3) % (N = 45). As noticed above, Equation 8 requires the solubility data in the mono-solvents at each temperature. When the equivalent terms from Equation 5 were replaced with $\log X_{1,T}$ or $\log X_{2,T}$ after training by two solubility data at 25 and 45ºC, the solubility of NAP at other compositions of PEG 200 and water could be predicted with the MRD of 34.5 (± 23.0) % (N = 51). The appearance of Equation 8 is slightly complex, however, the readers could be referred to an Excel file published online to predict the solubility of drugs after providing the input data. The file could be downloaded free of charge from: 

http://onlinelibrary.wiley.com/doi/10.1002/jps.22589/suppinfo.

**Figure 1:**
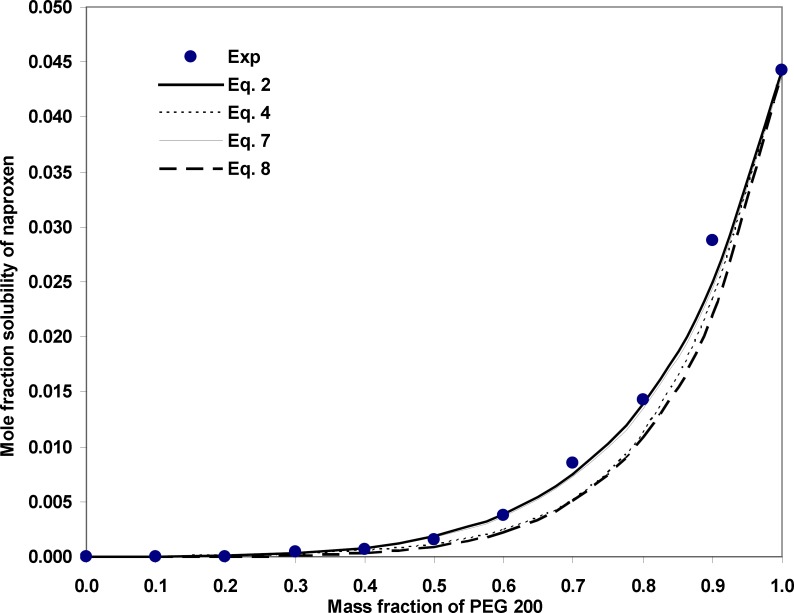
Experimental and calculated solubilities of NAP using different models in various solvent compositions of PEG 200 + water mixtures at 35ºC

**Figure 2:**
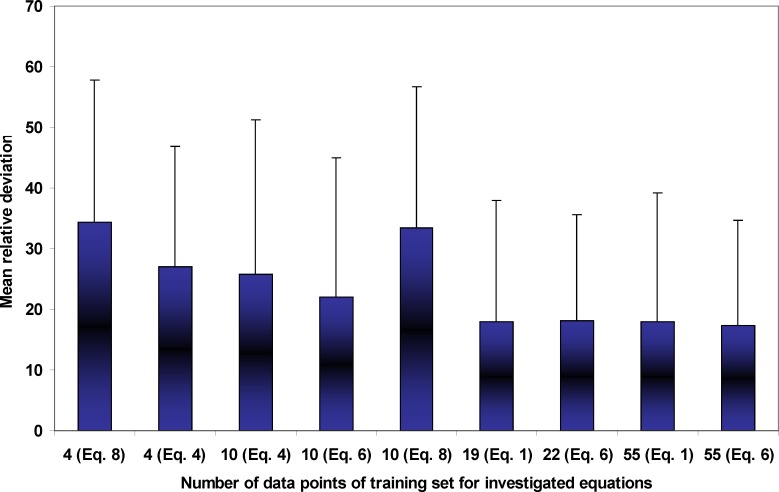
Relationship between number of employed data points and the MRD values for the predicted solubilities using different predictive models

Figure 1 illustrates the experimental and calculated solubilities of NAP in various solvent compositions of PEG 200 + water mixtures at 35ºC as a sample graph. As it is evident from the figure, due to the very wide range of the solubility from 6.94 x 10^-6^ to 4.43 x 10^-2^, the differences in the deviations between experimental solubilities and the calculated points are not visible. As expected the predicted solubilities using Equation 8 possess the largest deviations from experimental points which is also confirmed by MRD values and the best fit to the experimental solubilities belong to Equations 1 and 6. From a practical point of view, we prefer to predict the solubility data of a drug in the solvent systems without using experimental data which is not accessible at the present time. An alternative method is to reduce the number of required experimental data points to be used in the training process of the models. As a general rule, the more data points in the training set, the more accurate predictions are expected, however, collecting experimental solubility data is costly and time consuming. So, there should be a balance between expected MRD for predicted solubilities and the number of required data points. Figure 2 shows the relationship between number of employed data points and the MRD values for the predicted solubilities using different predictive models discussed in this work. Concerning Equation 1, which is specific for solubility of NAP in PEG 200 + water mixtures, there is no significant improvement in the prediction capability of the model after training with 19 or 55 data points. Therefore, training the model using all data points in the full range of solvent compositions at 25ºC is recommended. Then the solubility at other temperatures could be predicted employing solubility data in the mono-solvents at each temperature. Concerning the combined Jouyban-Acree model with van’t Hoff equation, *i.e*. Equation 6, training by 10 data points produces the prediction MRD of 22 %, increasing the number of training data points to 22 improves the MRD to 17 % and further increase in the number of training data points does not significantly change the MRD value. Therefore, for this version, training using whole data set at the lowest and highest temperatures is recommended. The trained model is able to predict the solubility of NAP in all possible compositions at various temperatures with the prediction error of ~ 17 %. The required input data for solubility prediction using Equation 4 is the solubility of NAP in the mono-solvents at each temperature of interest and the produced MRD is ~ 26 %. One could replace these data points with the equivalent terms from the van’t Hoff equation (trained by two data points) and reduce the number of required data points to 4. For this method the expected prediction error is ~ 27 %. Prediction of drug solubility in pharmaceutical cosolvents + water mixtures at various temperatures is one of the interesting methods for a pharmaceutical chemist. As shown in Figure 2, there is no significant difference between predicted solubilities employing 4 and 10 data points. Therefore, if the prediction error of ~ 35 % is accepted, Equation 8 could be the best predictive model, since it covers more cosolvents in comparison with the Equation 4 which covers only PEGs + water mixtures. It should be noted that PEGs could be used for other purposes in the pharmaceutical industries such as a dissolution rate enhancer and preparation of novel drug delivery systems.

*Thermodynamic analysis of NAP solubility*

According to van’t Hoff analysis, the apparent standard enthalpy change of solution (∆_soln_*H*°) for non-electrolyte drugs is obtained by using the mean harmonic temperature (*T*_hm_ is 308.0 K in the present case) according to Equation 9 (19).

${\left(\frac{{\partial \mathrm{lnx}}_{2}}{\partial (\frac{1}{T}-\frac{1}{T_{\mathrm{hm}}})}\right)}_{P}=-\frac{{∆}_{\mathrm{soln}}H^{{}^{\circ}}}{R}$

Equation (9)

where, *R* is the universal gas constant (8.314 J mol^–1^ K^–1^). On the other hand, the apparent standard Gibbs energy change for the solution process (∆_soln_*G*°) of non-electrolyte drugs considering the approach proposed by Krug *et al.* is calculated at mean harmonic temperature by means of:

${∆}_{\mathrm{soln}}G^{{}^{\circ}}=-{\mathrm{RT}}_{\mathrm{hm}}\times \mathrm{intercept}$

Equation (10)

in which, the intercept used is the one obtained in the analysis by treatment of ln *x*_2_ as a function of 1/*T* – 1/*T*_hm_. Finally, the apparent standard entropic change for solution process (∆_soln_*S*°) is obtained from the respective ∆_soln_*H*° and ∆_soln_*G*° values by using:

${∆}_{\mathrm{soln}}S^{{}^{\circ}}=\frac{\left({∆}_{\mathrm{soln}}{\mathrm{SH}}^{{}^{\circ}}-{∆}_{\mathrm{soln}}G^{{}^{\circ}}\right)}{T_{\mathrm{hm}}}$

Equation (11)

Table 2 summarizes the apparent standard thermodynamic functions for experimental dissolution process of NAP in all PEG 200 + water cosolvent mixtures. In order to calculate these thermodynamic quantities some methods of uncertainties propagation were used (20). It is found that the standard Gibbs energy of solution is positive in all cases as expected because the mole fraction is always lower than the unit and thus, its logarithmic term is negative, and therefore, standard Gibbs energy will be a positive quantity. _soln_*G* values diminish from neat water to neat PEG 200. Thermodynamic quantities obtained in neat water are similar to those reported previously in the literature for the same drug at 303.0 K, i.e. Δ_soln_*H*° = 21.3 kJ mol^-1^ and Δ_soln_*S*° = - 29.0 J mol^-1^ K^-1^ (3).

**Table 2:** Thermodynamic quantities for the dissolution process of naproxen in various mass fractions (*m*_1_) of PEG 200 (1) + water (2) mixtures at 308.0 K (35 ºC).

***m***_1_	_soln_***G*° /kJ mol**^-1^	_soln_***H*° /kJ mol**^-1^	_soln_***S*° /J mol**^-1^** K**^-1^	***T*** _soln_***S*° /kJ mol**^-1^	_H_	_TS_
0.000	30.5 (0.8)	20.5 (0.8)	-32 (2)	-9.9 (0.5)	0.674	0.326
0.100	26.2 (0.7)	73.0 (2.9)	152 (7)	46.8 (2.2)	0.610	0.390
0.200	23.9 (0.6)	72.5 (2.9)	158 (8)	48.6 (2.3)	0.599	0.401
0.300	20.1 (0.5)	47.9 (1.9)	90 (4)	27.8 (1.3)	0.633	0.367
0.400	18.5 (0.5)	52.2 (2.1)	109 (5)	33.7 (1.6)	0.608	0.392
0.500	16.6 (0.4)	38.4 (1.5)	71 (3)	21.8 (1.0)	0.638	0.362
0.600	14.4 (0.4)	30.4 (1.2)	52 (2)	15.9 (0.8)	0.656	0.344
0.700	12.6 (0.3)	45.8 (1.8)	108 (5)	33.2 (1.6)	0.580	0.420
0.800	10.9 (0.3)	46.3 (1.9)	115 (5)	35.4 (1.7)	0.566	0.434
0.900	9.6 (0.2)	47.1 (1.9)	122 (6)	37.5 (1.8)	0.557	0.443
1.000	8.2 (0.2)	59.0 (2.4)	165 (8)	50.9 (2.4)	0.537	0.463

The apparent enthalpy and entropy of solution are positive in all cases, therefore the process is always endothermic and driven by entropy. In different way to Gibbs energy of solution, _soln_*H* and _soln_*S* values increase from neat water to the mixtures of 0.10 or 0.20 in mass fraction of PEG 200 and they decrease beyond this mixture composition to the mixture of 0.60 in mass fraction of this cosolvent. 

With the aim to compare the relative contributions by enthalpy ( _H_) and by entropy ( _TS_) toward the solution process, Equations 12 and 13 were employed, respectively (21):

$\xi_{H}=\frac{\left|{∆}_{\mathrm{soln}}H^{{}^{\circ}}\right|}{\left|{∆}_{\mathrm{soln}}H^{{}^{\circ}}\right|+\left|{T∆}_{\mathrm{soln}}S^{{}^{\circ}}\right|}$

Equation (12)

$\xi_{\mathrm{TS}}=\frac{\left|{T∆}_{\mathrm{soln}}S^{{}^{\circ}}\right|}{\left|{∆}_{\mathrm{soln}}H^{{}^{\circ}}\right|+\left|{T∆}_{\mathrm{soln}}S^{{}^{\circ}}\right|}$

Equation (13)

From Table 2, it follows that enthalpy is the main contributor to standard Gibbs energy of solution process of NAP in all the systems studied and thus the energetic factor predominates. 

The cosolvent action may be related to the breaking of the ordered structure of water by hydrogen bonding around the non-polar moieties of the drug that increases both the enthalpy of and the entropy of the system. Above in mixture 0.10 in mass fraction of PEG 200, the apparent enthalpy lowering is the driving force that enhances NAP solubility.

**Figure 3:**
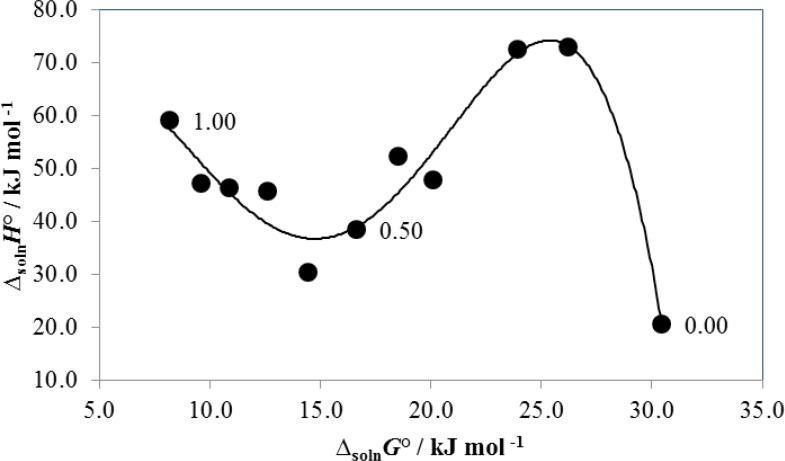
∆_soln_*H*° vs. ∆_soln_*G*° enthalpy-entropy compensation plot for the solubility of naproxen in PEG 200 + water mixtures at 308.0K. Points correspond to mass fraction of PEG 200 in the mixtures free of drug

Figure 3 shows that non-linear compensation is found when enthalpy-Gibbs energy coordinates are evaluated. In fact, the observed behavior could be treated as a quartic regular polynomial. Accordingly, from neat water to the mixture with 0.10-0.20 in mass fraction of PEG 200 a variant negative slope is found. According to the literature (3, 5) this result implies entropy-driving in the solution process and could be interpreted as the destroying of “icebergs” present near to the non-polar moieties of the drug as a consequence of the PEG 200 addition to the mixtures. From 0.20 to 0.60 in mass fraction of PEG 200 a positive slope is obtained and therefore, enthalpy-driving is obtained in this region. Normally, this result is interpreted as a consequence of favorable solute-solvent interactions increasing. Finally, from 0.60 in mass fraction of PEG 200 to neat PEG 200 a negative slope is obtained again. In this case the entropy-driving of the solution process could be interpreted in terms of the high degree of movement freedom in PEG polymer mixtures. 

## Conclusions

Measured solubility data in this work extends the available solubility database of pharmaceuticals in mixed solvent systems (22) providing useful information for pharmaceutical and related industries. A general cosolvency model using Jouyban-Acree model and partial solubility parameters proposed in a previous study, can be used to predict the solubility of NAP in PEG 200 + water at different temperatures with employing only four solubility data in the mono-solvents. As a general rule, the more accurate predictions are obtained with more input data. Since collecting more data points is not feasible in some situations, a pharmaceutical chemist should make a balance between expected prediction error and the number of experimental data to be collected. Using the Jouyban-Acree model the thermodynamic properties were calculated for naproxen in PEG 200 + water mixtures at different temperatures. In the other way, non-linear enthalpy-entropy compensation is found for the dissolution process in these mixtures. Thus, entropy-driving is found in water-rich and PEG 200 rich-mixtures and enthalpy-driving in mixtures of intermediate compositions. 
